# Supplementary material for: The Balance Hypothesis for the Avian Lumbosacral Organ and an Exploration of Its Morphological Variation
Source: Integr Org Biol. 2020 Aug 12;2(1):obaa024. doi: 10.1093/iob/obaa024 (PMC7751001; doi:10.1093/iob/obaa024)
Supplement: obaa024_Supplementary_Data [file obaa024_supplementary_data.zip › SupplementalInfo_LSOteamJuly2020.pdf]

Supplemental Information – Supplemental Table 1, Supplemental Figure 1, List of Museum Specimens, Supplemental Table 2, and notes on headings for "LSO Metrics" csv data file.

Supplemental Table 1. Summary statistics for the LSTC prominence and expansion ratio for the entire sample and for subsamples defined by locomotor ecology.

	Mean	Median	Standard deviation
**All (n=44)**			
LSTC prominence	0.156	0.157	0.054
Expansion ratio	4.173	3.904	1.204
**Perching (n=29)**			
LSTC prominence	0.171	0.170	0.055
Expansion ratio	4.168	4.05	0.971
**Not perching (n=15)**			
LSTC prominence	0.128	0.123	0.039
Expansion ratio	4.183	3.867	1.602
**Terrestrial (n=30)**			
LSTC prominence	0.157	0.163	0.04
Expansion ratio	4.067	3.786	1.291
**Not terrestrial (n=14)**			
LSTC prominence	0.155	0.132	0.077
Expansion ratio	4.401	3.96	0.998

Supplemental Figure 1. Contrast-enhanced CT scan of the lumbosacral organ (LSO) of *Taeniopygia guttata* demonstrates fluid space with the lumbosacral transverse canals (LSTCs).

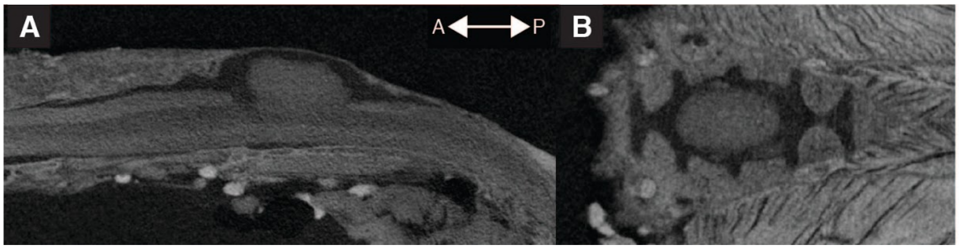

Supplemental Information - List of Museum Specimens

Acronyms: UWBM: Ornithology Collection, Burke Museum of Natural History and Culture; USNM: Division of Birds, Smithsonian National Museum of Natural History

Accipiter cooperii UWBM 65054

Aegolius funereus UWBM 44737

Aeronautes saxatalis UWBM 34156

Alectoris chukar UWBM 19808

Alisterus chloropterus UWBM 43544

Anas crecca UWBM 59245

Apus pacificus UWBM 44398, UWBM 44399, UWBM 46954, UWBM 46955, UWBM 46956

Butorides striatus UWBM 45810

Charadrius vociferus UWBM 88808

Cinclus mexicanus UWBM 50254

Colaptes auratus UWBM 19131, UWBM 32004, UWBM 32500, UWBM 87743, USNM 553938, USNM 610529

Colius striatus UWBM 38252, UWBM 38253, UWBM 52742, USNM 428106, USNM 491986, USNM 558540

Columba livia UWBM 76160

Coracia garrulus UWBM 56647

Cordeiles minor UWBM 65014

Corythaixoides concolor UWBM 52910

Cyanocitta stelleri UWBM 52505

Delichon urbica UWBM 47240

Eudyptula minor UWBM 57504

Falco sparverius UWBM 26276

Gallicolumba rufigula UWBM 43040

Gavia arctica UWBM 28567

Geococcyx californicus UWBM 34139

Hemiprocne mystacea UWBM 58752, UWBM 60348, UWBM 62344, USNM 559041, USNM 560829, USNM 572397

Jacana spinosa UWBM 21277

Megalaima lineata UWBM 64902

Merops bullockoides UWBM 57056

Nothoprocta perdicaria UWBM 65156

Pelecanoides urinator UWBM 60504

Phaethon rubricauda UWBM 68941

Pharomachus moccino UWBM 18395

Phaethornis guy UWBM 22394

Phoenicopterus chilensis UWBM 39909

Picus canus UWBM 51782

Podargus strigoides UWBM 76160

Podilymbus podiceps UWBM 30133

Progne subis UWBM 45892, UWBM 55860, USNM 501361, USNM 556284, USNM 610520

Puffinus bulleri UWBM 55371

Spheniscus humboldti UWBM 91277

Tachybaptus ruficollis UWBM 86353

Taeniopygia guttata (from laboratory colony)

Tyrannus verticalis UWBM 35770

Upupa epops UWBM 46408

Zonotrichia leucophrys (from laboratory colony)

Supplemental Table 2. LSTC Prominence, Expansion Ratio, and locomotor categorizations for each bird species. * indicates species means derived from multiple specimens; see supplemental file "UW_LSOteam_Apr2020_LSOmetrics.csv" for full data set. For locomotor categories, Y=yes and N=no. Note that only "Terrestrial" (bipedal in attached data file) and "Perching" were analyzed in the text.

Binomial	LSTC Prominence	Expansion Ratio	Fly	Perching	Float	Dive	Terrestrial
Accipiter cooperii	0.199	3.710	Y	Y	N	N	Y
Aegolius funereus	0.173	4.604	Y	Y	N	N	Y
Aeronautes saxatalis	0.084	3.766	Y	N	N	N	N
Alectoris chukar	0.129	4.056	N	N	N	N	Y
Alisterus choloropterus	0.191	4.552	Y	Y	N	N	Y
Anas crecca	0.140	3.307	Y	N	Y	N	Y
Apus pacificus	0.090*	3.867*	Y	N	N	N	N
Butorides striata	0.207	4.573	Y	Y	N	N	Y
Charadrius vociferous	0.156	4.402	Y	N	N	N	Y
Cinclus mexicanus	0.167	3.746	Y	Y	Y	Y	Y
Colaptes auratus	0.170*	3.307*	Y	Y	N	N	Y
Colius striatus	0.086*	2.997*	Y	Y	N	N	Y
Columba livia	0.203	5.150	Y	Y	N	N	Y
Coracias garrulus	0.189	6.141	Y	Y	N	N	N
Chordeiles minor	0.164	5.269	Y	Y	N	N	Y
Corythaixoides concolor	0.182	4.859	Y	Y	N	N	Y
Cyanocitta stelleri	0.237	3.677	Y	Y	N	N	Y
Delichon urbicum	0.093	4.050	Y	Y	N	N	Y

Binomial	LSTC Prominence	Expansion Ratio	Fly	Perching	Float	Dive	Terrestrial
Eudyptula minor	0.092	2.274	N	N	Y	Y	Y
Falco sparverius	0.191	3.614	Y	Y	N	N	N
Gallicolumba rufigula	0.159	5.130	N	Y	N	N	Y
Gavia arctica	0.111	3.144	Y	N	Y	Y	N
Geococcyx californianus	0.197	3.494	N	Y	N	N	Y
Hemiprocne mystacea	0.141*	4.925*	Y	Y	N	N	N
Jacana spinosa	0.132	3.328	Y	N	Y	N	Y
Psilopogon lineatus	0.163	3.557	Y	Y	N	N	Y
Merops bullockoides	0.126	3.826	Y	Y	N	N	Y
Nothoprocta perdicaria	0.114	3.130	N	Y	N	N	Y
Pelecanoides urinatrix	0.105	3.979	Y	N	Y	Y	N
Phaethon rubricauda	0.145	3.584	Y	N	Y	N	N
Pharomachrus mocinno	0.291	5.869	Y	Y	N	N	N
Phaethornis guy	0.091	3.413	Y	Y	N	N	N
Phoenicopterus chilensis	0.232	9.269	Y	N	Y	N	Y
Picus canus	0.163	3.128	Y	Y	N	N	Y
Podargus strigoides	0.336	5.651	Y	Y	N	N	N
Podilymbus podiceps	0.123	5.409	Y	N	Y	Y	N
Progne subis	0.124*	4.157*	Y	Y	N	N	Y
Ardenna bulleri	0.175	5.090	Y	N	Y	N	Y
Spheniscus humboldti	0.113	3.328	N	N	Y	Y	Y
Tachybaptus ruficollis	0.093	3.941	Y	N	Y	Y	N
Taenipygia guttata	0.107	2.364	Y	Y	N	N	Y
Tyrannus verticalis	0.182	4.315	Y	Y	N	N	N

Binomial	LSTC Prominence	Expansion Ratio	Fly	Perching	Float	Dive	Terrestrial
Upupa epops	0.170	4.967	Y	Y	N	N	Y
Zonotrichia leucophrys	0.138	0.140	Y	Y	N	N	Y

NOTES ON HEADINGS IN "LSO METRICS" DATA .csv FILE:

Genus, specific, Binomial, Epithet, Row -- These are all variously formatted species names.
LSTC - This is the "LSTC prominence" metric from the paper. It is calculated using a total of 11 sections, as described in the methods and in Figure 3a. The calculation is in the Matlab code provided with the submission. (referred to as "SOR" in matlab code)
ExRat - This is the "Expansion Ratio" metric from the paper. The calculation is in the Matlab code.
Fly, Perching, Float, Dive, Terrestrial - Locomotor categories (how they were assigned is described in the methods)
Perch_Tree, Terrestrial_Tree - These are the Fly and Perching columns reformatted to be used in the code that generate figures.
