# Supplementary material for: The Balance Hypothesis for the Avian Lumbosacral Organ and an Exploration of Its Morphological Variation
Source: Integr Org Biol. 2020 Aug 12;2(1):obaa024. doi: 10.1093/iob/obaa024 (PMC7751001; doi:10.1093/iob/obaa024)
Supplement: obaa024_Supplementary_Data [file obaa024_supplementary_data.zip › 017(2020) title page Stanchek .docx]

The balance hypothesis for the avian lumbosacral organ and an exploration of its

morphological variation

Kathryn E. Stanchak†1, Cooper French1, David J. Perkel*1, Bingni W. Brunton*1

1 - Department of Biology, University of Washington, Seattle WA 98195

* - These authors jointly directed this work.

† - corresponding author: [stanchak@uw.edu](mailto:stanchak@uw.edu)

Acknowledgements

The authors thank the Ornithology Collection at the Burke Museum of Natural History and

Culture and the Division of Birds at the Smithsonian National Museum of Natural History for

access to specimens. Sharlene Santana, Adam Summers, and Michelle Hickner provided access

and help with CT scanning. Cassandra Fieldson and Calvin Davis produced endocasts. Kimberly

Miller helped with soft tissue anatomical investigations, and Tanvi Deora provided helpful

discussion and references on insect inertial sensing. Alejandro Rico-Guevara and Tom Daniel

provided helpful comments and discussion on a draft of the manuscript.
